# Correction: Gorr, S.-U. Targeted Modification of the Antimicrobial Peptide DGL13K Reveals a Naturally Optimized Sequence for Topical Applications. *Microorganisms* 2025, *13*, 2355

**DOI:** 10.3390/microorganisms14010169

**Published:** 2026-01-13

**Authors:** Sven-Ulrik Gorr

**Affiliations:** Department of Diagnostic and Biological Sciences, University of Minnesota School of Dentistry, Minneapolis, MN 55455, USA; sugorr@umn.edu

In the original publication [1], there was a mistake in Figure 6 as published. The sample numbers for the left panel inadvertently included some samples from the right panel. The corrected Figure 6 appears below. There was a mistake in the legend for Figure 6 affecting the sample numbers reported and the use of control values. The correct legend appears below. 

The authors state that the scientific conclusions are unaffected. This correction was approved by the Academic Editor. The original publication has also been updated.

## Figures and Tables

**Figure 6:**
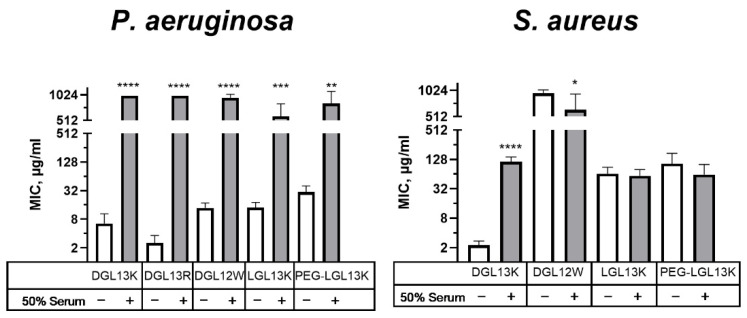
Relative activity of peptides in 50% serum. MICs of selected peptides were determined in the absence (open bars) or presence (shaded bars) of 50% serum for *P. aeruginosa* or *S. aureus*. Each sample pair was analyzed via unpaired Student’s *t*-test with Welch’s correction for different variances, as needed. MIC values outside of the measured range were set to 1000 µg/mL for calculation purposes. *P. aeruginosa*: Data from 1–8 independent experiments are plotted as means ± 95% confidence intervals. ** *p* < 0.002; *** *p* = 0.0002; **** *p* < 0.0001 (N = 2–15). *S. aureus*: Data from 2–10 independent experiments are plotted as means ± 95% confidence intervals. * *p* < 0.05; **** *p* < 0.0001 (N = 4–21). Some values obtained in the absence of serum were also included in earlier figures to aid in the comparison between different experimental conditions.
